# Carbon Sequestration by Preparing Recycled Cement, Recycled Aggregates, and Recycled Concrete from Construction and Demolition (C&D) Wastes

**DOI:** 10.3390/ma17205020

**Published:** 2024-10-14

**Authors:** Jing Luo, Rong Huang, Junjie Wang, Yi Zhang

**Affiliations:** Department of Civil Engineering, Tsinghua University, Beijing 100084, China; gcyzb@mail.gufe.edu.cn (J.L.); hr23@mails.tsinghua.edu.cn (R.H.); zhang-yi@tsinghua.edu.cn (Y.Z.)

**Keywords:** carbon sink, C&D wastes, carbon sequestration, recycled cement, recycled aggregates, recycled concrete

## Abstract

As the world’s largest producer of construction waste, China’s recycling and related policies are of the biggest concern to the world. However, the effective disposal and reuse of this waste has become an important issue since currently China still has a very low recycling ratio compared to developed countries, and most of the waste concrete was only simply broken and used as low-grade recycled aggregates for subgrade cushion, cement stabilized crushed stone, and filler wall. In this paper, a concrete cycle model focusing on how to effectively recycle and utilize waste concrete is put forward to prepare high quality recycled concrete, especially through a series of technical means, such as effective separation, carbon sequestration, and reactivation. Producing high quality recycled concrete can not only replace traditional concrete but also effectively reduce the consumption and waste of raw materials. What’s more, the calculation results show a potential of significantly carbon sink; for every ton of recycled cement produced, the CO_2_ emission could be reduced by 0.35–0.77 tons compared to ordinary Portland cement, corresponding to a reduction of 47%–94%; and for every ton of recycled concrete produced, the CO_2_ emission could be reduced by 0.186 tons compared to normal concrete. A yearly CO_2_ sequestration of 1.4–3.08 gigatonnes could happen if the ordinary Portland cement could be replaced by the recycled cement around the world. Taking the currently accumulated construction and demolition (C&D) wastes globally, the production of recycled cement, recycled aggregates, and recycled concrete could induce a significant carbon sink in the world.

## 1. Introduction

Large amounts of construction and demolition (C&D) waste are generated in many countries due to continuing urbanization on a global scale. From 1990 to 2018, the amount of C&D waste generated every year in the US increased by 342%, reaching more than 600 million tons of C&D waste in 2018 [1]. Australia generated 5.58 million tons of C&D waste in 2009, England generated 77.38 million tons in 2010, EU-27 countries generated 857.16 million tons in 2010 [2], and Seoul generated 24 million tons in 2021 [3]. In contrast, China generated 2.19 billion tons in 2011 [2] and 3.21 billion tons in 2021 [4]. The annual global C&D waste could easily double by 2025 [5]. As the world’s largest construction site, China also produces one of the largest construction wastes in the world. With the acceleration of urbanization and the continuous advancement of infrastructure construction, construction waste in China has become an important issue in urban environmental governance and resource recycling [6,7,8,9,10,11,12]. The process of dismantling construction waste generates exhaust gases such as CH_4_, N_2_O, SO_2_, and CO in addition to mainly CO_2_ [13]. However, these wastes are not worthless, and through effective treatment and utilization, they can be transformed into valuable resources that contribute to sustainable development.

The production of cement concrete is a major source of CO_2_ emissions and a major consumer of natural resources. Currently, cement is produced at 4 gigatonnes a year in the world [14]. Cement production emits 2.3 billion tons of CO_2_ per year, which accounts for 6.5% of the global carbon emissions [15]. In order to reduce CO_2_ emissions during the production of cement and concrete, researchers have proposed the utilization of poultry litter ash and calcined marl as supplementary cementitious materials [16,17,18]. This approach has the potential to effectively reduce CO_2_ emissions. Furthermore, the natural carbon sequestration effect of waste concrete could be an important way to reduce carbon emissions. Theoretically, almost all of the CO_2_ emissions from the decomposition of carbonates in the raw materials for producing cement can be reabsorbed by the hydrated cement pastes in concrete. However, the waste concrete needs to be grounded into small particles, especially the old cement paste particles, in order to ease the diffusion of CO_2_ into the pastes [15]. Waste concrete can be transformed into recycled materials with carbon sequestration capacity through special treatment. In this way, large amounts of CO_2_ previously emitted can be sequestered, reducing the level of carbon emissions in the atmosphere. This is of great significance in the fight against global climate change [15,19,20,21,22,23].

Carbon-based materials are now widely used as new and advanced materials in fields such as industry and biomedicine [24], and in the building materials industry, the carbonation of waste concrete has been the subject of intensive study in recent years. It has been estimated that carbonated recycled cement, recycled coarse aggregate, and recycled fine aggregate all exhibit an extraordinary carbon capture capacity, permanently fixing 190 kg/t, 20 kg/t, and 7.9 kg/t of CO_2_, respectively [25,26]. This technique represents a more effective and economical approach to utilizing waste concrete for carbon sequestration than alternative methods such as chemical absorption. Furthermore, the application of carbonated waste concrete products in the concrete industry has the potential to reduce CO₂ emissions by over 9% [27].

Despite the existence of a greater number of studies on the subject of recycled aggregate, recycled cement, and recycled concrete emissions and captured CO₂ [28,29,30], it is still the case that the full calculation of recycled concrete emissions and captured CO₂ remains less well-developed. In light of the aforementioned limitations, this paper puts forth a concrete recycling model for the production of high-quality recycled concrete. The model emphasizes the effective recycling of waste concrete through a series of technical means, including effective separation, carbon sequestration, and reactivation. The production of high-quality recycled concrete has the potential to not only replace traditional concrete but also to effectively reduce the consumption and waste of raw materials. Effective treatment and utilization of construction waste in China is of great significance for achieving sustainable development [31,32]. Not only does it help to conserve and recycle natural resources, but it also reduces carbon emissions through the carbon sequestration treatment on waste concrete. This requires the joint efforts of the government, enterprises, scientific research institutions, and the public to jointly promote the development of Chinese construction waste recycling. Only in this way can we protect our planet and create a better future for future generations.

## 2. Establishment of Concrete Cycle Model

The concrete cycle model considers the process starting from the demolition of old concrete structures and recycling of waste concrete, producing recycled aggregates and recycled cement, which are then reused to produce the recycled concrete. During preparing the recycled aggregates and recycled cement, carbonation treatment is applied to obtain CO_2_ sequestration and enhance their properties [33,34].

### 2.1. Recycled Cement

**Figure 1:**
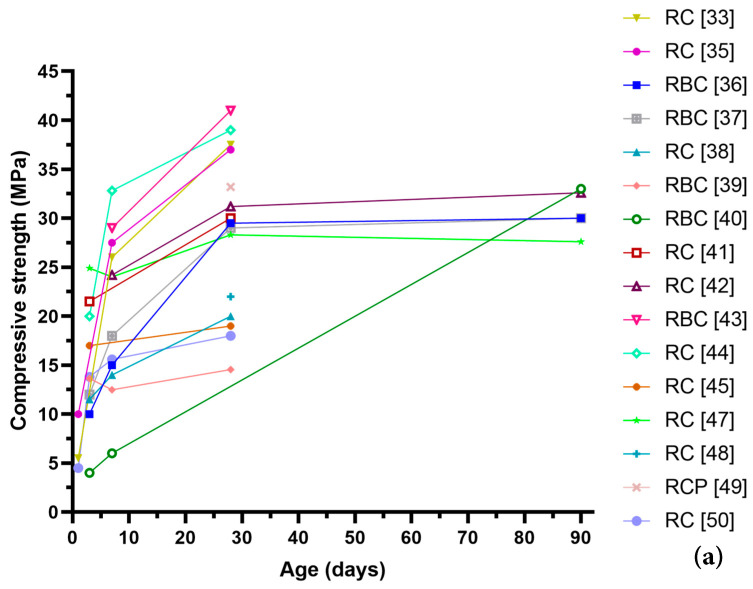

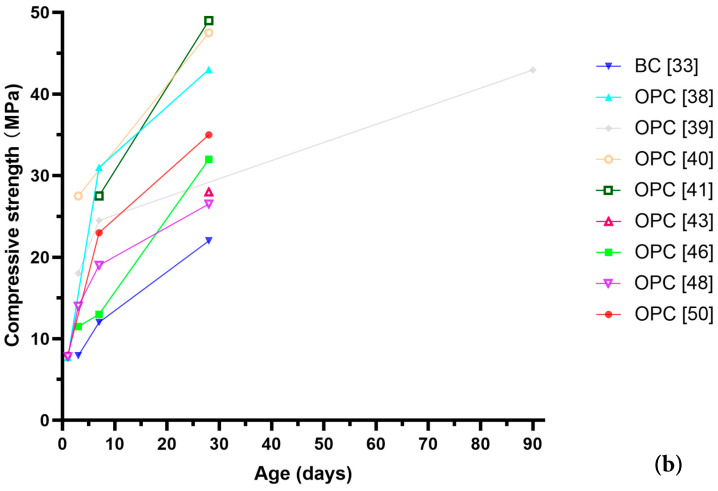
Comparison of reported compressive strengths of (**a**) recycled cement pastes and (**b**) ordinary cement pastes in different literature (RC—Recycled Cement, RBC—Recycled Blended Cement, RCP—Recycled Concrete Powder, OPC—Ordinary Porland Cement, BC—Blended Cement). (data from [33,35,36,37,38,39,40,41,42,43,44,45,46,47,48,49,50]).

**Figure 2:**
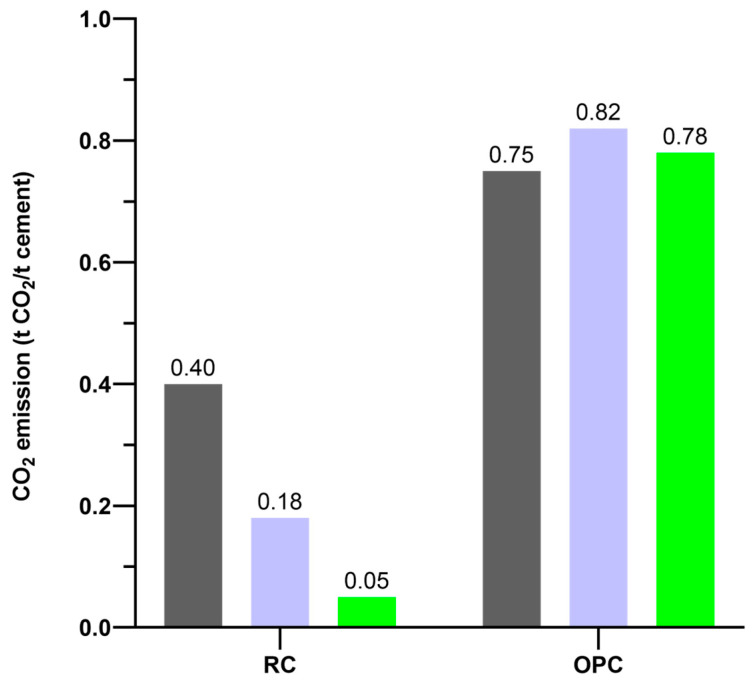
Comparison of reported CO_2_ emissions for producing per ton of recycled cement (RC) and ordinary Portland cement (OPC). (grey [40], purple [46], green [52]) (data from [40,46,52]).

Recycled cement uses the old cement paste obtained from waste concrete as the precursor, and then the old cement paste undergoes grinding, carbonation, and thermal activation. Through proper activation methods, including thermal treatment [35,36], the old cement paste powder can be transformed into recycled cement, which can be directly used to replace the ordinary Portland cement. Figure 1 shows the comparison of reported compressive strength of recycled cement pastes and ordinary Portland cement pastes at the curing ages of 1, 3, 7, 28, and 90 days with a water-binder ratio from 0.5 to 0.72 in different literature [33,35,36,37,38,39,40,41,42,43,44,45,46,47,48,49,50]. It can be seen that the pastes made from 100% recycled cement, recycled blended cement, or recycled concrete powder could still reach the same strength level as the ordinary Portland cement pastes [33,35,36,37,38,39,40,41,42,43,44,45,46,47,48,49,50]. Different types of cement paste could have different optimum activation temperatures [37]. Generally, the thermal activation temperature is below the decomposition temperature of calcite in the powder, and the suggested activation temperature is 650 °C from our previous work [37,51]. Most importantly, the CO_2_ emission from producing per ton of recycled cement is reported to be only 0.05–0.40 tons CO_2_ compared to the 0.75–0.82 tons CO_2_ for ordinary Portland cement as in Figure 2, corresponding to a 47%–94% reduction in CO_2_ emission depending on the technologies and activation methods applied [40,46,52]. Considering the yearly production of 4 gigatonnes of ordinary Portland cement in the world, the potential carbon sink is significant, corresponding to a CO_2_ sequestration of 1.4–3.08 gigatonnes every year. However, the recycled cement could still need some mineral or chemical additives to modify its workability, setting time, and physical properties [35,36,51].

### 2.2. Recycled Aggregates

The recycled aggregates include both fine and coarse aggregates. Recycled aggregates still have some old cement paste attached to the aggregate surface, in which there are a lot of pores and microcracks [34]. For recycled aggregates, they can also be used to directly replace 100% of the sand and stone (fine and coarse aggregates) in concrete through proper treatments, such as stripping off the attached cement paste, carbon sequestration, surface coating, etc. [53,54,55]. However, without these treatments, recycled aggregates from simply breaking waste concrete can only be used to replace below 30% of natural aggregates without obviously compromising the physical properties of concrete. In addition, a simple breaking method could produce enough cement paste for preparing the recycled cement [56,57,58]. Research into the carbon sequestration of recycled aggregates has demonstrated their potential in enhancing the performance of building materials and promoting carbon neutrality. Carbonation could generate the calcites and fill in the pores and microcracks, thus enhancing the properties of recycled aggregates and adhesion between recycled aggregates and the cement paste when preparing recycled concrete. The formation of carbonated precipitated CaCO_3_ whiskers can facilitate the development of a robust bond between the aggregate and the new matrix, thereby enhancing the overall bond strength in the transition zone [59]. The carbonated aggregate has the capacity to desorb sufficient quantities of moisture to facilitate internal curing of the concrete, thereby counteracting the adverse effects of drying shrinkage in hardened concrete [60,61]. A substantial body of empirical evidence attests to the efficacy of concrete prepared with carbonated recycled fine aggregate, which can attain the same strength as that prepared with natural sand [62]. Furthermore, the utilization of carbonated recycled coarse and fine aggregates allows for the production of C40 concrete without modifying the cement admixture [63]. By optimizing the carbonation conditions and processes, the performance of recycled aggregates can be effectively improved, and the effective fixation of CO_2_ can be achieved during this process. Carbon sequestration is the most cost-effective way to improve the properties of recycled aggregates, and paste stripping is the most effective way to obtain the recycled aggregates with similar properties as the pristine aggregates, but it usually requires more energy. Combined paste stripping and subsequent carbon sequestration could be the best option for preparing high quality recycled aggregates and, at the same time, providing enough precursor materials for producing recycled cement [64,65,66,67]. Future studies could further explore the mechanisms by which carbon sequestration affects the performance of recycled aggregates, as well as how to apply this to practical construction projects in order to promote the sustainable development of building materials.

### 2.3. Recycled Concrete

Here the recycled concrete is made of 100% recycled aggregates (fine and coarse), 100% recycled cement, water, and other required additives if needed. In an actual engineering project, in order to obtain the required properties of recycled concrete, the recycled aggregates and recycled cement could be used to partly replace the ordinary aggregates and cement, in which case the recycled concrete should be called partial recycled concrete.

### 2.4. Carbon Sequestration

The carbon sequestration was applied to enhance the properties of recycled aggregates by forming calcites to fill the pores and microcracks in the old cement paste attached to the surface of recycled aggregates. In recycled cement, the calcite formed through carbonation can stay intact after thermal activation and contribute to the rehydration process of recycled cement. During carbon sequestration, high concentrations of CO_2_ and favorable environmental conditions are provided in a carbonation chamber to accelerate the reaction between CO_2_ and the related phases in concrete [10,68,69]. Natural exposure to the atmosphere for a long time could still be satisfied. Our previous study [33,70] reported enhanced microstructure of recycled cement paste and ITZ between recycled aggregates and cement paste with carbon sequestration on the production of recycled cement and recycled aggregates.

The fundamental principle of carbon sequestration through the carbonation of cement-based materials primarily involves the reaction of carbon dioxide with the hydration products of cement, such as calcium hydroxide and hydrated calcium silicate, as well as unhydrated cement particles, to form calcium carbonate. During this process, the reaction of calcium hydroxide to form calcium carbonate results in an increase in volume by 11.8%, thereby improving the pore structure of recycled aggregates and enhancing their mechanical properties. Through carbon sequestration, the performance of recycled aggregates can be improved, reducing their porosity and water absorption rate and also enhancing the mechanical and durability performance of recycled concrete [71]. In most cases, cement paste in concrete has been hardened for years; the carbonation process happened as Equations (1)–(4):

Ca(OH)_2_ + CO_2_ → CaCO_3_ + H_2_O (1)

C─S─H (hydrated calcium silicate) + CO_2_ → CaCO_3_ + SiO_2_·nH_2_O (silica gel) (2)

2CaO·SiO_2_ (C_2_S)/3CaO·SiO_2_ (C_3_S) + CO_2_ + H_2_O → CaCO_3_ + SiO_2_·nH_2_O (silica gel) (3)

(4) $$3\mathrm{CaO}\cdot {\mathrm{Al}}_{2}O_{3}\cdot 3\mathrm{Ca}\overset{–}{S}O_{4}\cdot 32H_{2}O\;(\mathrm{AFt})\;/\;3\mathrm{CaO}\cdot {\mathrm{Al}}_{2}O_{3}\cdot \mathrm{Ca}\overset{–}{S}O_{4}\cdot 18H_{2}O\;(\mathrm{AFm})\;+\;{\mathrm{CO}}_{2}\;\;\to \;{\mathrm{Al}}_{2}O_{3}\cdot {\mathrm{nH}}_{2}O\;(\mathrm{aluminum}\;\mathrm{hydroxide}\;\mathrm{gel})\;+\;{\mathrm{CaCO}}_{3}\;+\;\mathrm{Ca}\overset{–}{S}O_{4}\cdot 2H_{2}O\;+\;H_{2}O$$

### 2.5. Concrete Cycle Model

**Figure 3:**
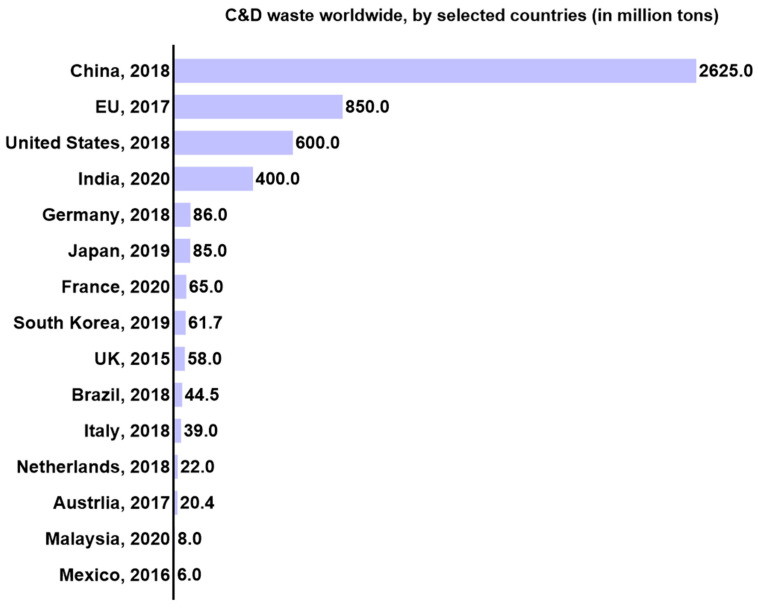
The weight of C&D wastes in main countries worldwide (data source from [72,73,74,75,76,77,78,79,80]).

**Figure 4:**
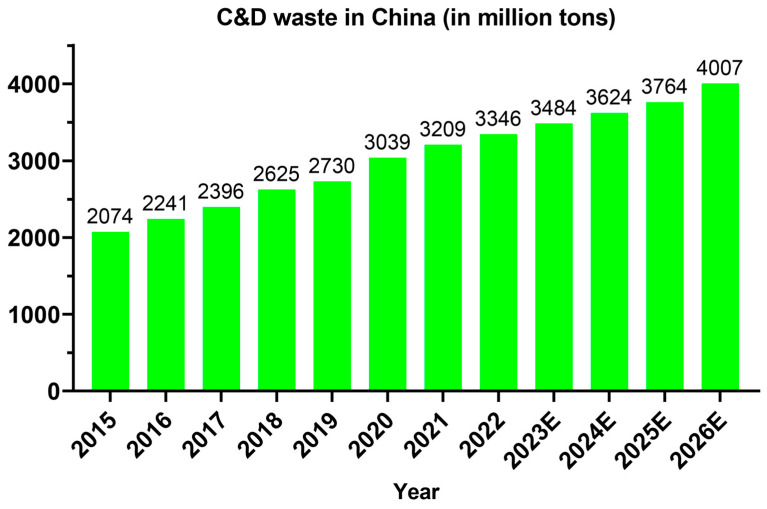
The trends of C&D wastes in China (The numbers in 2023E to 2026E represent years, and the E stands for estimated. Data from [81]).

**Figure 5:**
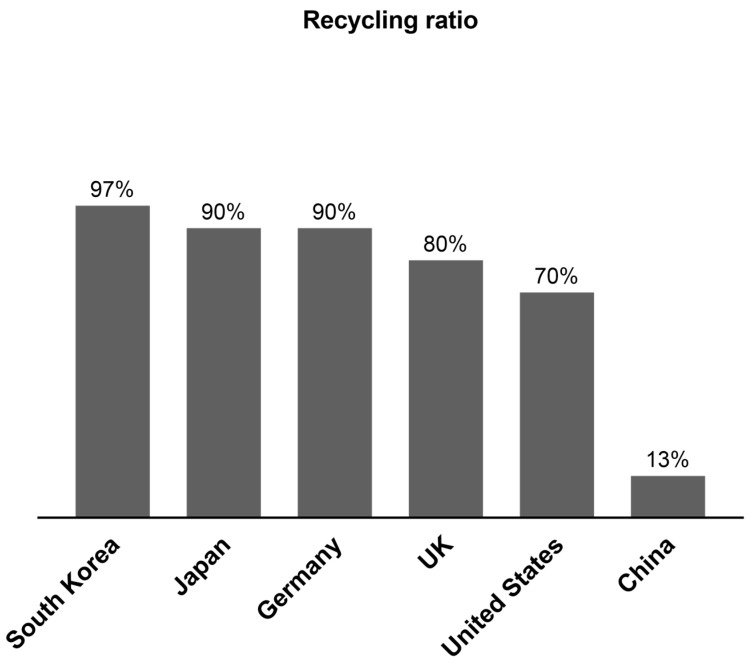
Comparison of recycling ratios of C&D wastes in different countries at the year 2020 (data from [82]).

**Figure 6:**
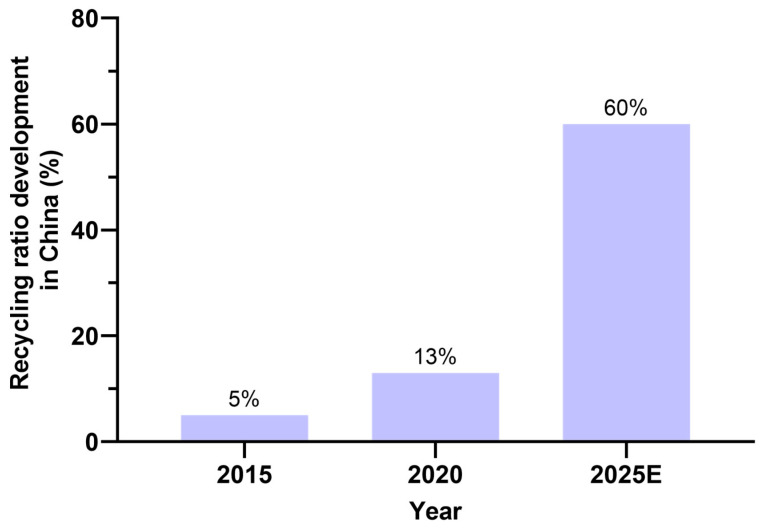
Remarkable development trend of the recycling ratios of C&D wastes in China (data from [33]).

Figure 3 shows the weight of C&D waste generated yearly in different countries worldwide during recent years [72,73,74,75,76,77,78,79,80]. It can be seen that the majority of the C&D waste generated in the world is in China. It is estimated that China generates 30–40% of the C&D wastes of the whole world every year [81]. From Figure 4, it can be seen that the C&D wastes generated yearly in China have been consistently increasing and are expected to reach 4 billion tons in 2026 [82]. The urgent situation is that China generates most of the C&D waste in the world but has a too low recycling ratio compared to developed countries. In 2020, China’s recycling ratio was only 13%, which is too little compared to other countries, such as the United States 70%, the UK 80%, Germany and Japan 90%, and South Korea 97%, as shown in Figure 5 [83]. Although currently China has a very low recycling ratio of C&D wastes, the Chinese government has set a target to reach a recycling ratio of C&D wastes at 60% by the end of the year 2025 [84]. Figure 6 shows the remarkable development trend on the recycling ratios of C&D wastes in China: only 5% in 2015, 13% in 2020, and a must-reach target of 60% in 2025. With this huge developing trend continuing, China will soon reach the same level as the developed countries. Effective recycling of construction waste can save natural resources. During the construction process, a huge amount of raw materials such as cement, sand, stone, etc. are consumed. By recycling and disposing of these C&D wastes, the need for new raw materials can be reduced, which in turn reduces the extraction and consumption of natural resources [85]. Not only does this help protect the planet’s finite resources, but it also provides a sustainable path for economic development. Recycling of resources is an important goal of sustainable development. Through professional technical means, waste concrete can be decomposed into various valuable materials, such as recycled aggregates, recycled cement, etc. These recycled materials can be used in new construction projects, forming a closed-loop resource recycling model. This not only reduces the accumulation of waste but also provides a sustainable source of building materials for the construction industry.

**Figure 7:**
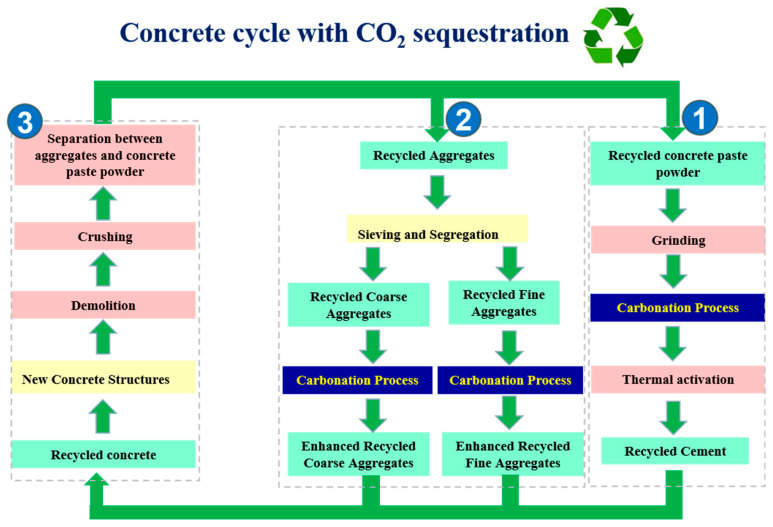
Production of recycled cement and recycled concrete with enhanced CO_2_ sequestration in concrete cycle.

Here, a concrete cycle map with enhanced CO_2_ sequestration is put forward as shown in Figure 7, which consists of 3 sections: Section 1 for producing recycled cement from recycled concrete paste powder, Section 2 for enhancing the recycled aggregates by carbonation, and Section 3 for producing and separating recycled aggregates and recycled concrete paste powder from waste concrete. The concrete cycle mainly includes producing recycled concrete with recycled aggregates and recycled cement, and carbon sequestration was applied to obtain CO_2_ sequestration in recycled aggregates and recycle cement. The waste concrete was recycled and crushed after demolition, and then paste powder and aggregates were separated. The recycled concrete paste (old cement paste) powder was used for producing recycled cement through grinding, carbonation, and thermal activation. The recycled aggregates were divided into recycled fine and coarse aggregates and enhanced with carbonation. The recycled cement and recycled aggregates were then used to produce the recycled concrete and start the next round of the concrete cycle. Since the phases in concrete that can be carbonated mainly exist in cement pastes, with a continuous concrete cycle, carbon sequestration could carbonate all the available phases, and at that time, the carbon sequestration step could not be necessary. On the other hand, additives or new ordinary cement could be added to produce the recycled concrete in order to obtain the required physical properties, and then the new phases introduced could be carbonated further. However, it should be noted that the long-term performance and durability of the prepared recycled concrete should be validated before the large scale of application in actual engineering projects.

## 3. Calculation of CO_2_ Emissions in Concrete Cycle Model

The model and calculation process of CO_2_ emissions in the concrete cycle is considered step by step in Figure 7 and consists of 3 sections, namely the production of recycled cement, recycled aggregates, and recycled concrete and its recycling at the final stage. Each section considers the difference in CO_2_ emissions between recycled concrete and normal concrete. The final difference of CO_2_ emissions for producing per ton of recycled concrete and normal concrete is calculated.

When one cycle in Figure 7 is considered, the calculation can be made based on the CO_2_ emissions for per ton of recycled aggregates and recycled cement, and then finally for per ton of recycled concrete produced with 100% recycled aggregates and recycled cement. The model can be listed as follows:

E_R_ = E_1_ + E_2_ + E_3_ (5)

where E_R_ is the CO_2_ emissions of producing per ton of recycled concrete from the concrete cycle model as in Figure 7. E_1_, E_2_, and E_3_ are the CO_2_ emissions in Section 1, Section 2, and Section 3 in Figure 7.

In Section 1, the process of producing recycled cement powder includes grinding, carbonation, and thermal activation. Grinding and thermal activation produce CO_2_ emissions, while carbonation induces CO_2_ sequestration.

E_1_ = E_G_ − E_C1_ + E_T_ (6)

where E_G_, E_C1_, and E_T_ are the CO_2_ emissions or sequestration of grinding, carbonation, and thermal activation for producing recycled cement.

In Section 2, the process mainly includes sieving and separation between recycled coarse (≥ 5 mm) and fine (< 5 mm) aggregates, and the carbonation process.

E_2_ = E_S_ − E_C2_ (7)

where E_S_ and E_C2_ are the CO_2_ emission or sequestration of sieving and carbonation for producing enhanced recycled aggregates through carbonation.

In Section 3, this section includes the mixing and preparation of recycled concrete from previously prepared recycled cement and recycled aggregates, demolition at the end of service, crushing, and separation between aggregates and concrete paste powder.

E_3_ = E_P_ + E_D_ + E_CR_ + E_SE_ (8)

where E_P_, E_D_, E_CR_, and E_SE_ are the CO_2_ emissions of preparation of recycled concrete, demolition, crushing, and separation between aggregates and concrete paste powder.

The main interest for us to consider the CO_2_ emissions in the concrete cycle is that what is the difference between the CO_2_ emissions for producing per ton of recycled concrete and normal concrete.

In E_1_, the main difference between producing the recycled cement and ordinary Portland cement is from the burning stage or thermal activation stage (E_T_) and the carbonation stage (E_C1_) in producing recycled cement. It can refer to the calculation for CO_2_ emissions of ordinary Portland cement. Take a typical advanced cement plant with an annual cement production of 846,320 tons in China. For example, the annual CO_2_ emission is 553,512 tons, which accounts for 0.66 tons of CO_2_ per unit ton of cement production [86]. These emissions include 34% CO_2_ from burning of fuel or thermal energy, 61% CO_2_ from decomposition of carbonates in the raw materials of cement, and 5% CO_2_ from electricity usage [87]. Taking 0.66 tons for producing per ton of ordinary Portland cement in an advanced cement plant in China, the calculations are as follows:

(1) For E_T_, the burning energy needed for the activation of recycled cement is only 45% of that for ordinary Portland cement (650 °C for recycled cement [36,37] vs. 1450 °C for ordinary Portland cement). Then the CO_2_ from burning of fuel for producing per ton of recycled cement is around 0.66 × 34% × 45% = 0.10 tons.
(2) For E_C1_, in hardened cement paste or recycled cement, the carbonates are mainly calcites, and the decomposition of them can only happen above 650 °C [36,37], which means there are hardly any CO_2_ emissions from the decomposition of carbonates in the raw materials of recycled cement. On the contrary, as shown in Figure 7, in the carbonation step before the thermal activation step of recycled cement, there could be a CO_2_ sequestration of 9% by weight of recycled cement [52] in the final recycled cement product. Then the CO_2_ emission from carbonation of raw materials per ton of recycled cement is −9% × 1 = −0.09 tons.
(3) The CO_2_ emission from electricity used for recycled cement can be assumed to be the same as that of ordinary Portland cement, which accounts for 0.66 × 5% = 0.033 tons per ton of recycled cement.

By combining the 3 parts of the CO_2_ emissions together, the total CO_2_ emissions for producing one ton of recycled cement is 0.10 − 0.09 + 0.033 = 0.043 tons, which was reduced by 93%, or 0.617 tons, compared to 0.66 tons as of the CO_2_ emission for ordinary Portland cement in Section 1. Then, the difference in E_1_ is a reduction of 0.617 tons of CO_2_ emissions.

In E_2_, there is the first sieving and segregation step, and then the carbon sequestration step. E_S_ can be taken as the same for both normal aggregates and recycled aggregates. When producing the machine made sand and normal coarse aggregates from stone, the procedures are the same as when producing recycled aggregates from waste concrete. E_C2_ is the benefit with CO_2_ sequestration in recycled aggregates since they usually contain cement pastes on surfaces, which can be carbonated.

(1) The sieving and segregation step is similar to the production of coarse and fine aggregates from the stone. Since the river sand has become scarce in most places in China, the fine aggregates made from stone have been used for normal concrete mix design. Thus, the CO_2_ emissions for the first step (sieving and segregation) in Section 2 are taken as the same as ordinary concrete, and thus the exact values were not reported and compared here.
(2) For the carbon sequestration step (E_C2_), the reported CO_2_ sequestration in recycled aggregates through carbonation, including both recycled coarse aggregates and recycled fine aggregates, varied between 0.4% and 3.5% by weight of recycled aggregates (i.e., 0.004-0.035 tons of CO_2_ sequestration with an average of 0.02 tons per ton of recycled aggregates) depending on the attached cement paste content on the surface, corresponding to an increase in calcium carbonates of 1%–8% in recycled aggregates [34]. Others reported a capacity of 65% absorption of the CO_2_ emitted originally from the cement in the cement paste [88]. In general, the recycled aggregates contain 30%–35% cement pastes [89] (original cement accounting for a weight of around 80% of hydrated cement paste), which corresponds to CO_2_ sequestration of 1×30%×80%×0.66×65% = 0.103 tons per ton of recycled aggregates.

In summary, compared to normal concrete, by considering reducing the CO_2_ emissions during the additional labor applied for the carbon sequestration, the recycled aggregates for recycled concrete could still have a CO_2_ sequestration of at least 0.1 tons per ton of recycled aggregates through the procedures in Section 2. Then, the difference in E_2_ is a reduction of 0.1 tons of CO_2_ emissions. This value varies with the amount of attached cement paste on the surfaces of recycled aggregates. If there are no attached cement pastes, this value is 0, and this carbonation step is not needed.

In E_3_, the procedures include the preparation of recycled concrete, the completion of the new concrete structures made of recycled concrete, demolition at the end of the service life, and crushing and separation between aggregates and concrete paste powder. The E_P_, E_D_, and E_CR_ can be taken as the same for both normal concrete and recycled concrete since the mixing and casting of concrete, demolition, and final crushing are also needed for normal concrete. The final crushing for normal concrete is usually used for low quality recycled aggregates for road cushions or cement-stabilizing-gravel. The enhanced recycled aggregates could reach the same grade level as normal gravels and can be used as the full replacement of normal gravels. The main difference is E_SE_, in which the cement waste powder needs to be separated from the recycled aggregates in order to obtain the precursor for producing recycled cement, which will be considered for the increased CO_2_ emissions in this step.

(1) The procedures from preparation of recycled concrete to demolition at the end of the service life for recycled concrete could be taken as similar as the ordinary concrete, since the same steps have to be conducted. The only difference could be the mixing and preparation of different types of concrete, i.e., recycled concrete and normal concrete. Recycled concrete reported in this study is made of 100% recycled cement and 100% recycled aggregates, the preparation of which could cause slightly different CO_2_ emissions from ordinary concrete. The mixing of recycled cement could be more difficult than ordinary Portland cement, but the workability of recycled cement paste could be modified with the addition of additives, such as industrial waste GGBS [35,36]. The recycled concrete made with recycled aggregates and recycled cement only may not have the same workability as ordinary concrete, and superplasticizer could be needed for mixing recycled concrete to reach the same workability as ordinary concrete. Since both additives and superplasticizers were also often used in ordinary concrete, the CO_2_ emissions in these steps for both recycled concrete and ordinary concrete can be taken as similar, and the exact values are not reported and compared here.
(2) The most important part of recycling the waste concrete and transforming them into the recycled aggregates and recycled cement is the final step of Section 3, i.e., the crushing and separation between aggregates and concrete powder. The crushing step can be taken as the same as producing the coarse and fine aggregates from the stone mined from hills. The crucial step is the separation between recycled aggregates and recycled cement paste powder. Since the recycled cement paste powder is the precursor for producing recycled cement, the purity is essential for the properties of recycled cement. One emerging separation technology is magnetic separation, and the energy consumption is about 0.36 MJ/t [89]. Taking the average EU CO_2_ emission intensity from electricity production value 83 kg/MJ, the CO_2_ emission for the separation step is 83 × 0.36 = 29.88 kg/t = around 0.03 tons for per ton of recycled cement. It should be noted that, in order to obtain a higher content of recycled cement from waste concrete, the simple crushing and separation step as in Figure 7 is far from enough. Additional procedures, such as grinding, microwave treatment, high voltage electrical pulse, etc. [90], should be applied to remove the cement paste from the recycled aggregates. These additional steps will cause more CO_2_ emissions, which can be calculated from the electricity used for different technologies. The most recommended paste stripping technology by us is the microwave treatment. Since this technology has yet to be used on a large scale in industry, the CO_2_ emissions were not reported here.

By comparing the CO_2_ emissions for recycled concrete and ordinary concrete in Section 3 (E_3_), the CO_2_ emission is higher in recycled concrete, and the increased CO_2_ emission is 0.03 tons per ton of recycled cement.

In summary, for a typical concrete mix (both normal concrete and recycled concrete) with 0.19 tons of cement and 0.74 tons of aggregates for per ton of concrete (remaining 0.07 tons of water for hydration and workability), the CO_2_ emission of per ton of recycled concrete produced or recycling use of per ton of waste concrete from Figure 7 is much lower than that of the normal concrete, and the reduced CO_2_ emission from the procedures of the 3 sections in Figure 7 can be calculated as: 0.617 × 0.19 (Section 1) + 0.1 × 0.74 (Section 2) − 0.03 × 0.19 (Section 3) = 0.186 tons. That is to say, for every ton of recycled concrete produced, the CO_2_ emission can be reduced by 0.186 tons compared to normal concrete. Taking the expected China’s C&D wastes in 2023 for further calculation, the expected amount is 3484 million tons. In C&D wastes generated in China, around 54% are waste concrete, accounting for 1881 million tons of waste concrete. If all this waste concrete can be recycled for producing the recycled concrete based on the concrete cycle in Figure 7, then around 350 million tons of CO_2_ emissions can be reduced for the single year 2023 from the concrete cycle. This is a huge amount of CO_2_ reduction, and if this cycle can also be applied around the world, then an obvious carbon sink could be reached. Since the C&D wastes generated in previous decades could still be pilled and could be used through our treatments and transformed into recycled cement, recycled aggregates, and recycled concrete. Considering the yearly CO_2_ sequestration of 1.4–3.08 gigatonnes could happen if the ordinary cement can be replaced by the recycled cement around the world, the construction from making normal concrete and recycled concrete with recycled cement could induce a further carbon sink globally.

Based on the assumptions of the model, a conceptually similar example for calculation was found in Xu et al. [91]. For Section 1, the carbon emission per ton of ordinary Portland cement is 0.7398 tons, and the carbon emission per ton of recycled cement is calculated as E_1_ = 0.1132 − 0.09 + 0.0370 = 0.0602 tons, so the difference of E_1_ is a reduction of 0.6796 tons of CO_2_ emissions. The second part is that the difference in carbon emissions between 1 ton of natural aggregate and 1 ton of recycled aggregate is E_C2_ = 0.103 tons. Finally, Section 3 states that each ton of recycled concrete will increase the carbon emission by 0.03 tons compared to each ton of ordinary concrete. If calculated according to this model, it can be seen that each ton of fully recycled concrete is 0.507 tons less carbon emission than ordinary concrete, which is 0.1541 tons more than Xu’s calculation. This is because of the consideration of the carbon sequestration link; if we add the third part of the recycled part of the fully recycled concrete, it can still reduce 0.1241 tons of CO_2_, which is very informative for the future reduction in CO_2_.

The proposed model is presented in a simplified manner, which precludes the incorporation of additional low-carbon materials as supplementary cementitious materials. It is anticipated that further research data will become available in the future, enabling the inclusion of this component in the calculations and, consequently, an enhancement of the model. Currently, there is a dearth of facilities for the complete recycling of carbonated waste concrete, which has resulted in a paucity of empirical data and may potentially lead to discrepancies between the calculated outcomes and the actual scenario. To address this, we intend to conduct experiments that emulate the real-world conditions as per the model, with the aim of comparing the calculated results with the experimental data. This will facilitate the calibration of the model to align with the actual circumstances, thereby enabling its utilization as a predictive tool for guiding future production decisions.

In addition, in order to achieve the effective CO_2_ reduction and utilization of waste concrete, it is necessary for the government, enterprises, and scientific research institutions to work together. The government should introduce relevant policies to encourage and support the recycling and treatment of construction waste. Enterprises should actively participate in C&D and apply advanced treatment technologies. Scientific research institutions should strengthen research to provide technical support and guidance for the resource utilization of construction waste. At the same time, public participation and education are crucial. Improving the public’s awareness and understanding of construction waste recycling, enhancing environmental awareness, and helping to form a good atmosphere for the participation of the whole society. In this way, the effective recycling and utilization of construction waste not only contribute to environmental protection but also contributes to the sustainable use of resources and promotes the green development of the economy.

## 4. Conclusions

In order to effectively recycle waste concrete, this paper proposes a concrete cycle model for the production of high-quality recycled concrete. The model takes the current global accumulation of C&D waste as an example and produces recycled cement, recycled aggregate and recycled concrete, and the following conclusions are drawn:

(1) This paper introduces a novel recycling model for waste concrete, which employs a suite of technical interventions, including effective separation, carbon sequestration, and reactivation, to produce high-quality recycled concrete. The model not only addresses the issue of waste management but also contributes to the global carbon sink by utilizing carbon sequestration techniques to fabricate recycled cement, recycled aggregates, and recycled concrete.
(2) Carbon sequestration technology fills the pores and microcracks on the surface of recycled aggregates by generating calcium carbonate, which effectively improves the performance of recycled aggregates and strengthens the adhesion between recycled aggregates and cement paste, providing a guarantee for the preparation of high-performance recycled concrete. In addition, the carbonation curing technology can improve the microstructure of the interface transition zone between recycled cement paste and recycled concrete, improve its mechanical properties and durability, and further enhance the quality of recycled concrete.
(3) The total CO_2_ emissions from the production of one ton of recycled cement are 0.617 tons lower than those of ordinary Portland cement. The potential for CO_2_ sequestration is estimated to be between 1.4 and 3.08 gigatonnes annually if the global cement industry were to transition from using ordinary Portland cement to recycled cement. For each ton of concrete using 0.19 tons of cement and 0.74 tons of aggregate, each ton of recycled concrete produced reduces 0.186 tons of carbon dioxide emissions compared to ordinary concrete.
(4) The circular utilization of construction waste as presented in this model offers a viable solution to the environmental challenges posed by the construction industry in China. The integration of effective separation, carbon sequestration, and activation technologies can lead to the production of high-quality recycled concrete, thereby reducing the environmental footprint of the construction sector and promoting a sustainable future.

## Data Availability

Dataset available on request from the authors.
